# Extensive Cochlear Otosclerosis: CT-MRI Correlation and the Diagnostic Value of Contrast Enhancement

**DOI:** 10.3390/diagnostics16182998

**Published:** 2026-09-16

**Authors:** Roberts Tumeļkāns, Cenk Eraslan, Arturs Balodis

**Affiliations:** 1 Institute of Diagnostic Radiology, Pauls Stradins Clinical University Hospital, LV-1002 Riga, Latvia; 2 Radiology Department, Ege University, 35040 Bornova, Turkey; 3 Department of Radiology, Riga Stradins University, LV-1007 Riga, Latvia; 4 Faculty of Medicine and Life Sciences, University of Latvia, LV-1004 Riga, Latvia

**Keywords:** otosclerosis, otospongiosis, cochlear, computed tomography, magnetic resonance imaging

## Abstract

Otosclerosis is a chronic disorder of the otic capsule. It is characterized by abnormal bone remodeling, which may extend beyond the fenestral region and involve the cochlea and other structures of the bony labyrinth. There are two main types of otosclerosis—retrofenestral (cochlear) and fenestral, the latter of which is much more common. We present multimodal imaging findings of extensive bilateral cochlear otosclerosis in a 59-year-old woman with chronic bilateral sensorineural hearing impairment, classified as grade 3 in the right ear and complete deafness in the left ear. High-resolution computed tomography (CT) demonstrated extensive grade 3 bilateral otospongiotic changes involving multiple cochlear turns and extending toward the vestibule, fissula ante fenestram, oval window, and the lateral semicircular canal. Pre-contrast T1-weighted magnetic resonance imaging (MRI) showed mild pericochlear hyperintensity, suggestive of otospongiotic bone remodeling. Matching post-contrast T1-weighted imaging findings corresponded with bilateral pericochlear and perilabyrinthine enhancement of the otic capsule, which is associated with findings compatible with an active otospongiotic bone remodeling. T2-weighted sequences showed preserved fluid signal within the labyrinth and no corresponding otic capsule abnormality. Although limited for directly depicting otospongiotic changes, the T2-weighted sequence was useful for assessing morphology and excluding alternative pathology. This case illustrates the complementary role of CT and MRI in demonstrating advanced active cochlear otosclerosis and draws attention to MRI findings that might otherwise be overlooked, especially those in post-contrast T1-weighted images.

**Figure 1:**
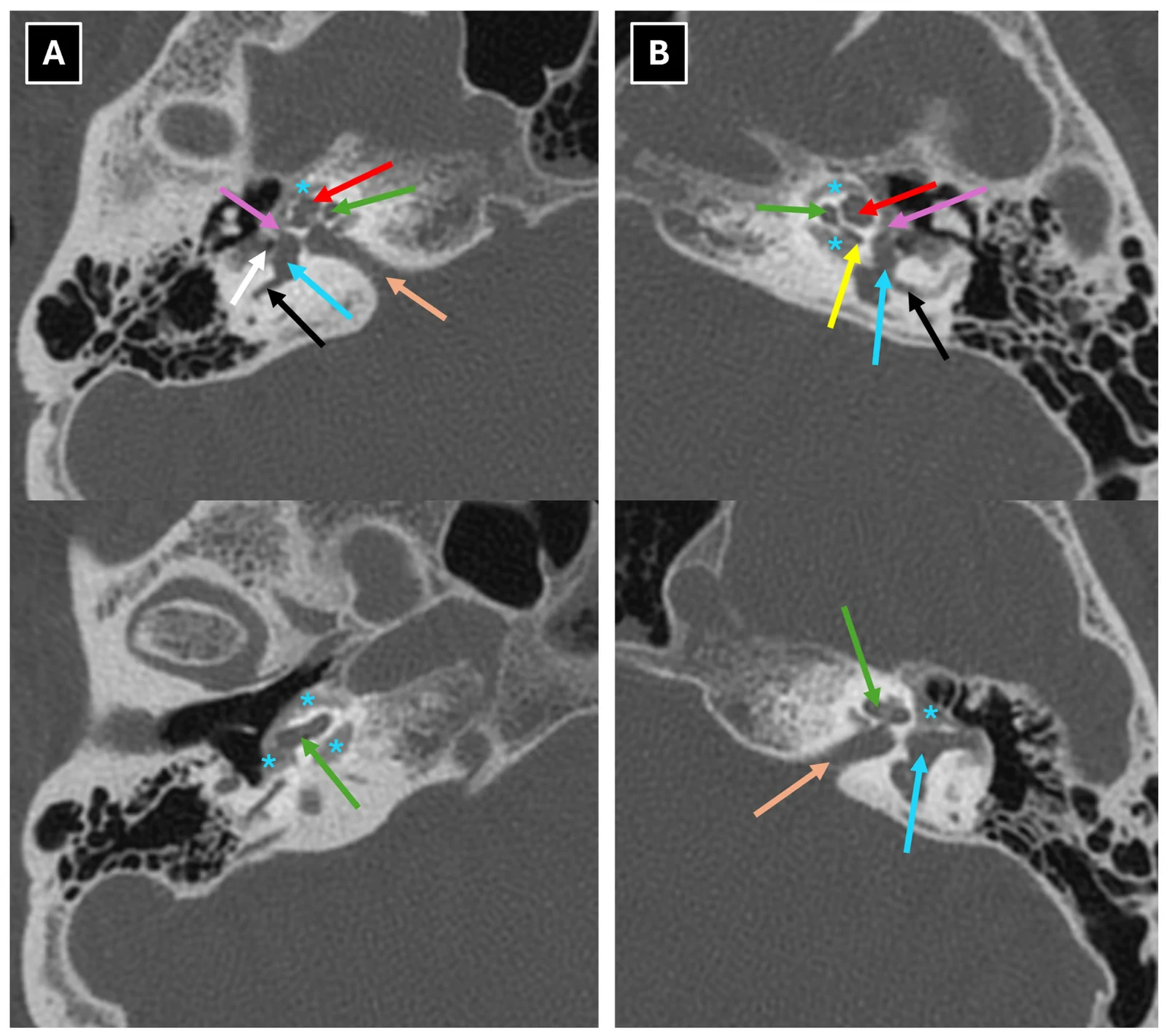
High-resolution computed tomography (CT) of a bilateral cochlear grade 3 otosclerosis in a 59-year-old woman. Axial images of the right (**A**) and left (**B**) temporal bones demonstrate extensive, relatively symmetric pericochlear hypoattenuating demineralization involving multiple cochlear turns and producing the characteristic “double-ring” appearance. Otospongiotic changes extend toward the vestibule, fissula ante fenestram, oval window, and lateral semicircular canal. The bilateral diffuse and confluent cochlear involvement corresponds to grade 3 otosclerosis according to the Symons–Fanning CT classification. An enlarged vestibule due to the otospongiotic changes can be seen. CT images were acquired with a “GE Revolution Maxima 64-slice” machine and reconstructed at 0.6 mm slice thickness. Black arrow—lateral semicircular canal; blue arrow—vestibule; blue asterisk—otosclerosis; green arrow—basal turn of cochlea; orange arrow—internal auditory canal/vestibulocochlear (VIII) nerve; pink arrow—fissula ante fenestram—the most common site of otosclerotic involvement; red arrow—middle turn of cochlea; white arrow—oval window; yellow arrow—cochlear window. The radiologic diagnosis of otosclerosis is based on high-resolution computed tomography [1]. Axial CT is considered the primary projection for the diagnosis of otosclerosis, as the relevant anatomical structures are best visualized on these images [2].

**Figure 2:**
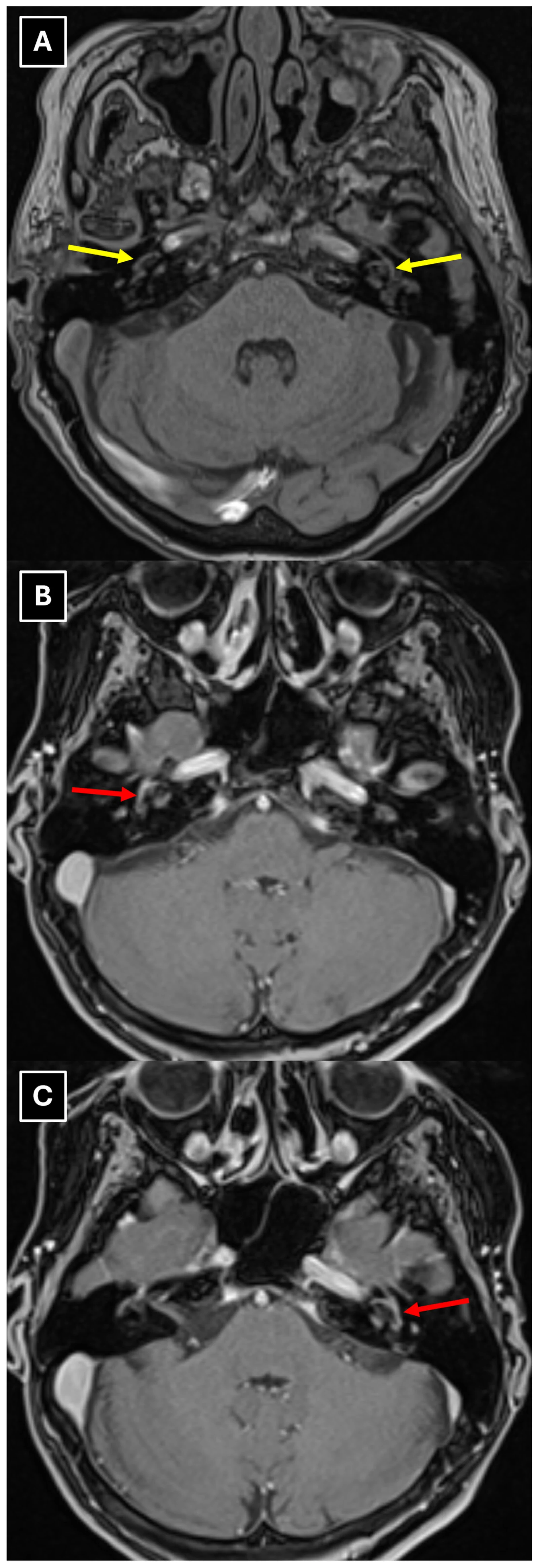
Brain magnetic resonance imaging (MRI) of bilateral cochlear otosclerosis in a 59-year-old woman. Axial pre-contrast (**A**) and matching post-contrast T1-weighted Volumetric Interpolated Breath-Hold Examination (VIBE) FS 1.0 mm images of the right (**B**) and left (**C**) temporal bones, selected at slightly different craniocaudal levels to optimally demonstrate the corresponding pericochlear abnormalities. Pre-contrast image (**A**) shows mild pericochlear hyperintensity, which is suggestive of otospongiotic bone remodeling. Post-contrast images (**B**,**C**) show bilateral pericochlear/perilabyrinthine enhancement of the otic capsule. The abnormal enhancement follows the osseous labyrinth rather than forming a discrete soft tissue mass, a pattern consistent with increased vascularity and findings compatible with an active otospongiotic bone remodeling. These findings correspond with the features seen in Figure 1. MRI images were acquired with a Siemens “MAGNETOM Sola” 1.5T system (Siemens Healthineers, Erlangen, Germany). Post-contrast images were acquired approximately 3–5 min after intravenous contrast administration. Yellow arrow—otospongiotic bone; red arrow—contrast enhancing otospongiotic bone. In many cases, particularly in patients with mixed or sensorineural hearing loss, MRI also has an important role [2]. Characteristic MRI findings in otosclerosis include a relatively increased T1 signal within the pericochlear and perilabyrinthine regions compared with the normally low signal of the surrounding tissue, as well as enhancement in these areas following contrast administration [2,3]. MRI findings might help in detecting otosclerosis in patients who initially undergo MRI for sensorineural hearing loss when CT is not available. A case series of 13 patients has been published in which MRI facilitated the recognition of previously undiagnosed otosclerosis [3]. Studies have also described the repeated use of MRI for evaluating the response to conservative pharmacological treatment [1,4]. Therefore, recognition of the MRI features of otosclerosis may provide specialists with broader opportunities for the clinical management of patients with otosclerosis.

**Figure 3:**
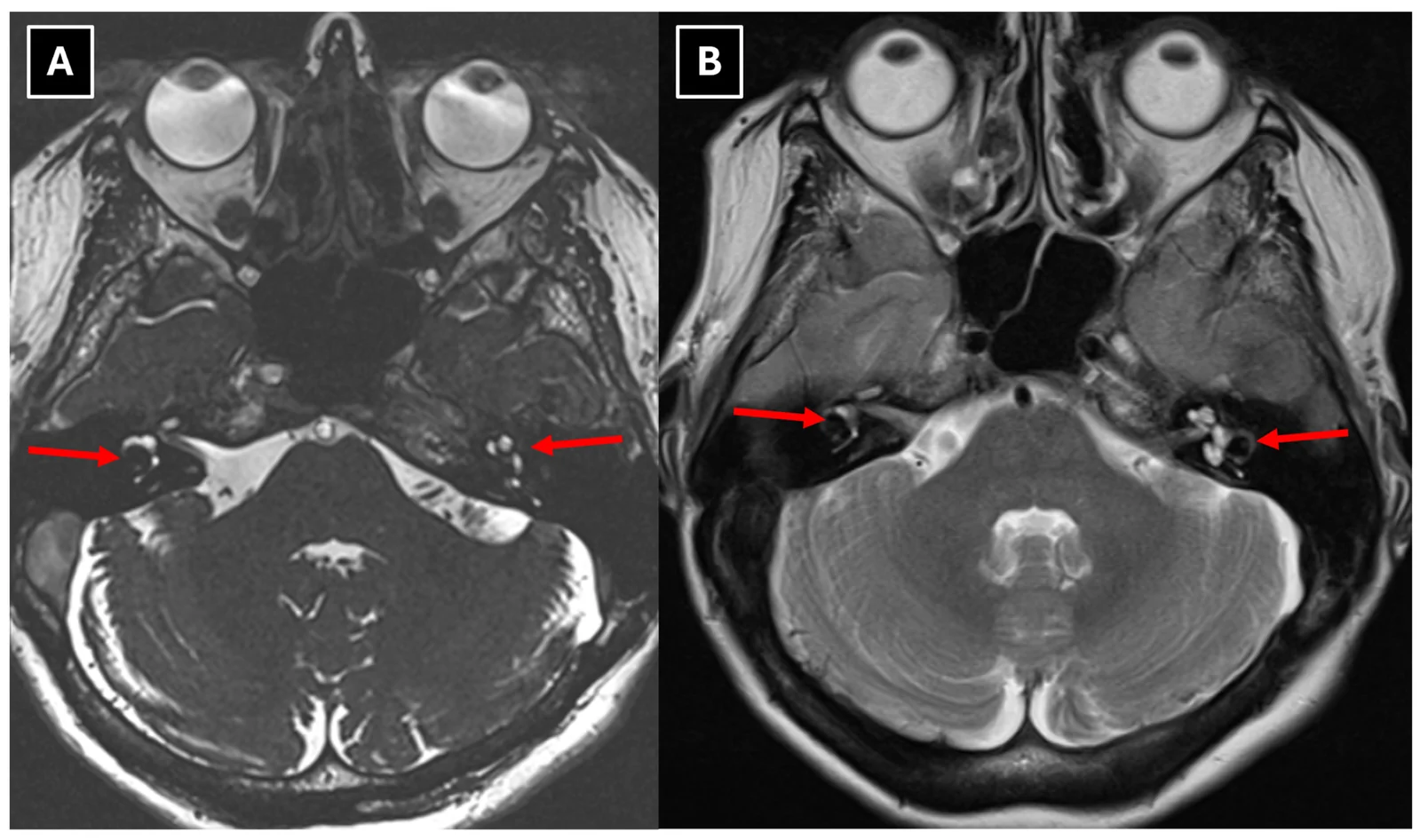
Brain MRI of bilateral otosclerosis in a 59-year-old woman. (**A**): Axial 3D T2-weighted Constructive Interference in Steady State (CISS) and (**B**): T2-weighted Turbo Spin Echo (TSE) images demonstrated preserved fluid signal within the cochlea, vestibules, and visualized semicircular canals, without evidence of labyrinthine obliteration. No corresponding T2-weighted abnormalities within the otic capsule can be seen. Red arrow—otic capsule. These T2-weighted sequences are of limited sensitivity for directly depicting otospongiotic changes of the otic capsule. However, they are useful for assessing labyrinthine morphology and excluding alternative retrocochlear pathology. Although the literature indicates that MRI findings in otosclerosis are less widely recognized [5], many authors emphasize their increasing importance in the evaluation of skull-base bone and inner ear pathology, with the T2 CISS sequence being particularly valuable because of its high contrast resolution [6]. MRI provides a better opportunity to assess the size of the cochlear canal, which may in turn be important for treatment planning and for predicting the outcome of surgical treatment [2].

## Data Availability

The original contributions presented in this study are included in this article; further inquiries can be directed to the corresponding author.

## Abbreviations

The following abbreviations are used in this manuscript:

CISS	Constructive Interference in Steady State
CT	Computed Tomography
MRI	Magnetic Resonance Imaging
TSE	Turbo Spin Echo
VIBE	Volumetric Interpolated Breath-Hold Examination
